# Comparative Study of Ultrasonic Vibration-Assisted Die-Sinking Micro-Electrical Discharge Machining on Polycrystalline Diamond and Titanium

**DOI:** 10.3390/mi15040434

**Published:** 2024-03-25

**Authors:** Cheng Guo, Longhui Luo, Zhiqiang Liang, Hao Li, Xiawen Wang, Bin Xu

**Affiliations:** 1 Guangdong Provincial Key Laboratory of Micro/Nano Optomechatronics Engineering, College of Mechatronics and Control Engineering, Shenzhen University, Shenzhen 518060, China; 2210295123@email.szu.edu.cn (L.L.); 2210295114@email.szu.edu.cn (H.L.); wangxiawen2022@email.szu.edu.cn (X.W.); binxu@szu.edu.cn (B.X.); 2 Shenzhen Key Laboratory of High Performance Nontraditional Manufacturing, College of Mechatronics and Control Engineering, Shenzhen University, Shenzhen 518060, China; 3 School of Mechanical Engineering, Beijing Institute of Technology, Beijing 100081, China; liangzhiqiang@bit.edu.cn

**Keywords:** ultrasonic, vibration, die-sinking, micro-EDM, PCD, titanium, diamond, discharge, MRR

## Abstract

Die-sinking micro-electrical discharge machining (micro-EDM) is a potential method used to fabricate intricate structures without complex electrode motion planning and compensation. However, machining efficiency and poor discharge states are still bottlenecks. This study conducted a comparative investigation into the impact of ultrasonic vibration on die-sinking micro-EDM of polycrystalline diamond (PCD) and pure titanium (TA2). By adjusting discharge parameters, this study systematically evaluated the influence of ultrasonic vibration on these two materials based on discharge waveforms, motion trajectories, effective discharge counts and groove profiles. At an open-circuit voltage of 100 V, ultrasonic vibration promotes die-sinking micro-EDM of PCD. However, when the open-circuit voltage increases to 200 V, ultrasonic vibration exhibits inhibitory effects in general. Conversely, for TA2, ultrasonic vibration shows a promoting effect at both voltages, indicating the differences of ultrasonic vibration-assisted die-sinking micro-EDM on PCD and TA2. For PCD, ultrasonic cavitation improves the discharge gap environment, accelerating the removal of discharge debris. For TA2, due to its poor thermal conductivity, ultrasonic cavitation acts to break the arc, accelerating heat transfer. These research findings provide guidance for ultrasonic vibration-assisted die-sinking micro-EDM in industrial applications.

## 1. Introduction

Micro-electrical discharge machining (micro-EDM), as a non-conventional material removal method, is widely applied in the processing of high-hardness and difficult-to-machine materials [1,2], especially for tiny parts [3,4,5]. Die-sinking micro-EDM, compared to micro-EDM milling [6], offers advantages such as high efficiency and absence of complex electrode compensation. However, its drawbacks include difficulties in chip evacuation and flushing due to small discharge energy levels and inter-electrode gaps. Therefore, the introduction of ultrasonic vibration is expected to address these issues. However, compared to the combination of conventional EDM and ultrasonic vibration, research on the integration of die-sinking micro-EDM and ultrasonic vibration remains limited. This study compares ultrasonic vibration-assisted micro-EDM of two typical difficult-to-cut industrial materials, polycrystalline diamond (PCD) and titanium.

PCD features a cobalt metal matrix with bonded diamond particles, endowing it with exceptionally high hardness and wear resistance. PCD is commonly used in cutting tools, durable electrodes and some vulnerable components, demonstrating promising applications in advanced materials processing [7,8]. However, it is difficult to machine PCD by mechanical methods [9]. Due to the poor conductivity of PCD, achieving a higher material removal rate (MRR) in micro-EDM of PCD material requires careful selection of appropriate machining parameters [10,11]. Yan et al. [12] designed a novel pulse generator that generated high-frequency pulse signals with an off-time period, enabling the reionization of dielectric in the machining gap, minimizing damage to the machined surface of PCD. Wang et al. [13,14] successfully manufactured a disk-shaped electrode and utilized the mobile arc generated by this electrode to expand the material removal range, significantly improving stability and processing performance.

Titanium and its alloys possess outstanding mechanical and physical properties, contributing to their extensive use in aerospace, engine turbine manufacturing and other fields. However, the low thermal conductivity and high toughness of titanium and its alloys make processing them challenging [15,16]. In micro-EDM of titanium, poor thermal conductivity, high hardness and significant chemical affinity result in frequent discharge failures [17,18]. When using different electrode materials and adjusting process parameters, MRR, surface roughness and electrode wear are generally enhanced with increases in current density and pulse duration [19,20,21].

As mentioned earlier, PCD and titanium alloys demonstrate low efficiency in EDM due to their unique material characteristics [22]. Many research findings [23,24,25,26] indicated that ultrasonic vibration induced a stirring effect and cavitation, aiding in debris removal and preventing material deposition on machined surfaces. The introduction of ultrasonic vibration significantly influenced the MRR, tool wear rate and taper angle of titanium alloy workpieces [27]. Wang et al. [28] induced vibrations in workpieces and effectively improved the efficiency of machining micro-deep holes in hard materials. M. Goiogana et al. [29] indicated that ultrasonic vibration-assisted processing in pulse mode can effectively improve surface roughness and uniformity. Employing ultrasonic vibration on electrodes also can significantly enhance processing efficiency [30]. B. Jabbaripour et al.’s research [31] on machining titanium alloy materials indicated that power supply parameters had an impact on the MRR and surface quality of titanium alloy materials. Therefore, in practical applications, appropriate adjustments and optimization are necessary to determine the optimal ultrasonic amplitude to achieve the best performance [32].

In the context of die-sinking micro-EDM, ultrasonic vibration can significantly impact chip evacuation, machining quality and process stability. However, the specific nature and extent of these effects could be influenced by variations in power supply parameters and material characteristics. Our research results contribute to a deeper understanding of ultrasonic vibration-assisted die-sinking micro-EDM at different discharge energies and on different materials.

## 2. Materials and Methods

### 2.1. Materials

In addition to PCD, this study selects TA2 (pure titanium) as another material. The dimensions of the PCD samples were 10 mm × 10 mm × 2 mm, with the PCD layer sintered onto a hard alloy matrix with a thickness of around 0.5 mm. The TA2 samples were 10 mm × 10 mm × 2 mm blocks. Prior to testing, the samples were mechanically polished and then cleaned using ultrasonic cleaning.

**Figure 1:**
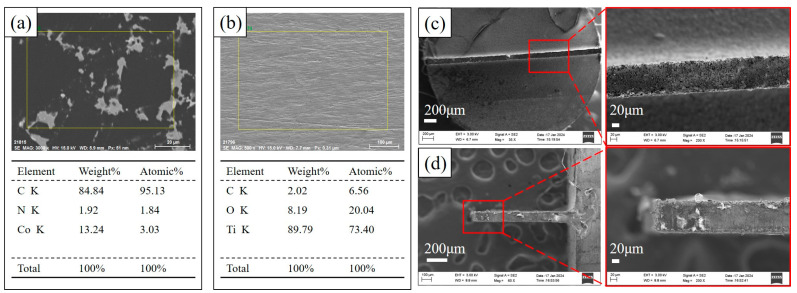
EDS spectra results of the sample materials and microscopic view of the electrode: (**a**) EDS of PCD; (**b**) EDS of TA2; (**c**) top view of the tool electrode; (**d**) side view of the tool electrode.

Figure 1 shows the EDS results of PCD and TA2 samples. In Figure 1a, the main elements in PCD are carbon (C) and cobalt (Co), where C primarily represents diamond particles, and Co serves as the binder. Figure 1b illustrates the elemental content on the surface of TA2. The tool electrode chosen for this experiment is W70Cu30 tungsten–copper alloy. Figure 1c,d shows that the tool electrode. The electrode tips have been wire-cut to fabricate the sheet tool electrode with the thickness of 100 μm.

### 2.2. Die-Sinking Micro-EDM Experimental Setup

**Figure 2:**
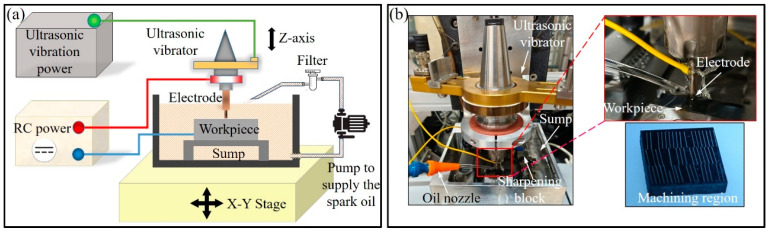
Experimental setup: (**a**) schematic diagram; (**b**) die-sinking micro-EDM setup and samples.

The schematic diagram of the experimental setup shown in Figure 2a includes an RC power supply, ultrasonic control power and EDM workstation. During die-sinking micro-EDM, spark oil serves as the dielectric, fully immersing the workpiece in spark oil throughout the process. The ultrasonic tool holder is mounted on a vertical platform, which moves according to servo commands. The experimental setup and machined samples are illustrated in Figure 2b. Near the processing area, a small fixture is installed to trim the worn electrode after each machining cycle. The ultrasonic device used in the experiment generates ultrasonic frequencies of 21,714 Hz. The ultrasonic power varies with the impedance between the workpiece and the electrode, while the ultrasonic amplitude ranges from 2 to 3 μm.

## 3. Results and Discussion

### 3.1. Ultrasonic Vibration-Assisted Die-Sinking Micro-EDM of PCD

**Figure 3:**
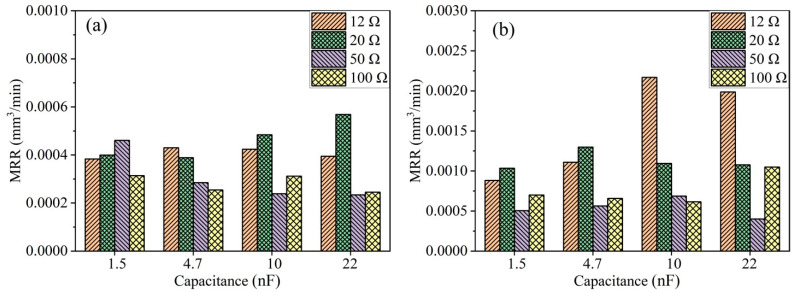
MRR of PCD at a machining voltage of 100 V: (**a**) without ultrasonic vibration; (**b**) with ultrasonic vibration.

As shown in Figure 3a, the MRR of PCD material in die-sinking micro-EDM, obtained under an open-circuit voltage of 100 V, exhibits significant differences under various discharge parameters. Without ultrasonic vibration, the MRR of PCD follows a trend of initial increase and subsequent decrease with the increase in discharge resistance, reaching its maximum at a discharge capacitance of 4.7 nF. However, with an increase in discharge resistance to 20 Ω, a distinctly different trend emerges, where MRR continues to increase with the rise in the capacitance. At a discharge resistance of 50 Ω, a trend opposite to that under a discharge resistance of 20 Ω is observed. The MRR continuously decreases with an increase in the capacitance.

The maximum MRR occurs with the combination of a capacitance of 22 nF and a discharge resistance of 20 Ω. For die-sinking micro-EDM, the discharge current significantly influences the MRR. At the largest capacitance, an increase in the discharge resistance leads to a decrease in the MRR. However, at a lower capacitance, such as 1.5 nF, the maximum MRR occurs at a discharge resistance of 50 Ω. The capacitance and the discharge resistance determine the maximum value of the single discharge energy and the feeding rate of the tool electrode.

As shown in Figure 3b, ultrasonic vibration-assisted die-sinking micro-EDM of PCD significantly enhances the performance, with overall MRR being higher than those without ultrasonic vibration, especially evident in the high-energy conditions. This result is consistent with the findings from Abdullah et al. in their research on ultrasonically assisted machining of tungsten carbide hard alloys. When the discharge peak current reaches 10 to 20 amperes, the material removal rate in ultrasonic vibration-assisted die-sinking micro-EDM is 4 to 5 times higher than that in conventional electrical discharge machining [23]. Inter-electrode ionization is more challenging to eliminate during high-energy discharges, which are also more prone to arc discharges. It becomes difficult for the dielectric fluid to enter the micrometer-scale inter-electrode gap, resulting in debris removal difficulty. Due to the vertical ultrasonic vibration, tiny bubbles in the inter-electrode spark oil undergo cavitation as a result of the repeated action of positive and negative acoustic pressure. These tiny bubbles experience expansion, compression and eventually collapse within the micro-scale discharge gap. On one hand, the cavitation effect improves the inter-electrode environment, allowing timely discharge debris removal; on the other hand, the cavitation effect induced by ultrasonic vibration can break the arc. In terms of electrode wear, there is a significant difference in electrode wear between ultrasonic vibration-assisted die-sinking micro-EDM and without ultrasonic vibration-assisted micro-EDM when machining PCD. Taking the combination of 12 Ω resistance and four sets of capacitors (1.5 nF, 4.7 nF, 10 nF and 22 nF) as an example, the electrode wear for ultrasonic vibration-assisted die-sinking micro-EDM is 30, 23, 29 and 23 μm, respectively. Meanwhile, the electrode wear without ultrasonic vibration-assisted die-sinking micro-EDM is 10, 20, 15 and 12 μm, respectively. Although ultrasonic vibration-assisted die-sinking micro-EDM achieves faster machining speeds, the electrode wear is also relatively high. The trend of electrode wear in ultrasonic vibration-assisted micro-EDM hole drilling observed in this study aligns with the findings of Atsutoshi HIRAO et al. [24].

**Figure 4:**
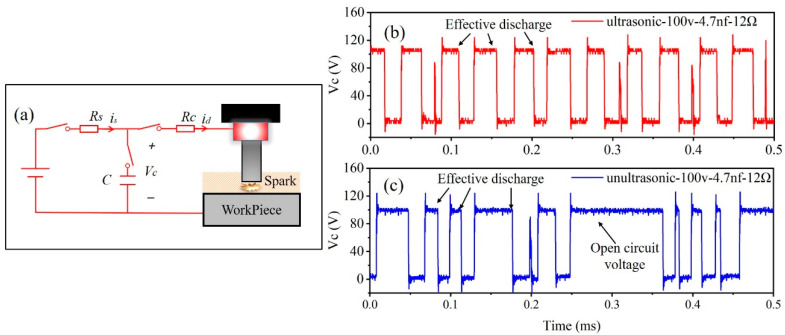
RC power supply and waveforms: (**a**) schematic diagram of the RC power supply circuit; (**b**) discharge waveforms with ultrasonic vibration; (**c**) discharge waveforms without ultrasonic vibration.

Figure 4a shows the schematic diagram of the RC power supply topology. Figure 4b,c represents the voltage waveform across the capacitor obtained under the discharge parameters of an open-circuit voltage of 100 V, a capacitance of 4.7 nF and a discharge resistance (RC) of 12 Ω. The waveform without ultrasonic vibration exhibits more open-circuit phenomena, with unstable time intervals between each pulse discharge and significantly fewer discharges per unit time compared to the counterpart with ultrasonic vibration.

**Figure 5:**
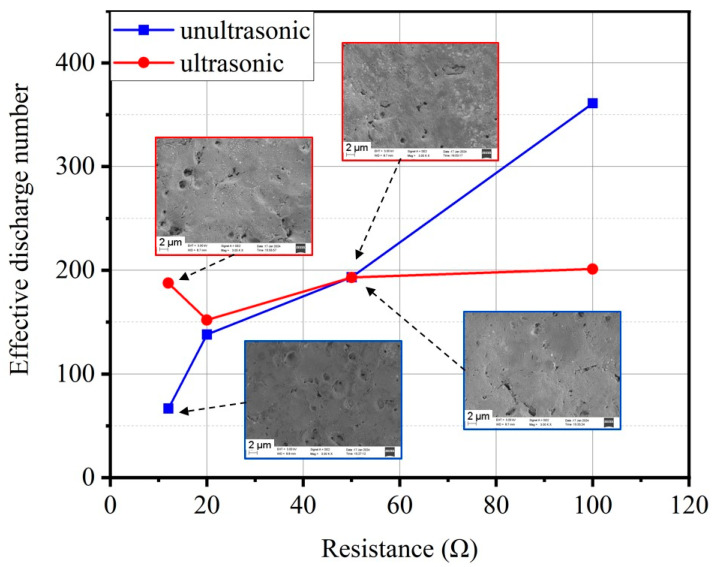
Effective discharge counts and SEM images for different discharge resistances under an open-circuit voltage of 100 V and a capacitance of 10 nF.

Figure 5 presents the average effective discharge counts and SEM images under an open-circuit voltage of 100 V and a capacitance of 10 nF with varying resistances. The average effective discharge counts are calculated over a 40 ms statistical period. Without ultrasonic vibration, the effective discharge counts increase with higher discharge resistance. At a discharge resistance of 12 Ω, the average discharge count is only 66. With increasing resistance, the average discharge count reaches 360 at a resistance of 100 Ω, accompanied by a noticeable reduction in surface craters. The introduction of ultrasonic vibration demonstrates a promotional effect on effective discharge counts for the case with low discharge resistance, aligning with the MRR results. When setting the discharge resistance to 12 Ω and 20 Ω, the effective discharge counts are enhanced. However, at a discharge resistance of 100 Ω, ultrasonic vibration exhibits a reduction in effective discharge counts compared to that without ultrasonic vibration. It is essential to note that ultrasonic vibration did not significantly improve the surface quality of the workpiece. SEM imaging reveals that discharge craters are primarily correlated with the discharge current, unrelated to ultrasonic vibration. This indicates that ultrasonic vibration has complex and varied effects on effective discharge counts in die-sinking micro-EDM under different power supply parameters.

**Figure 6:**
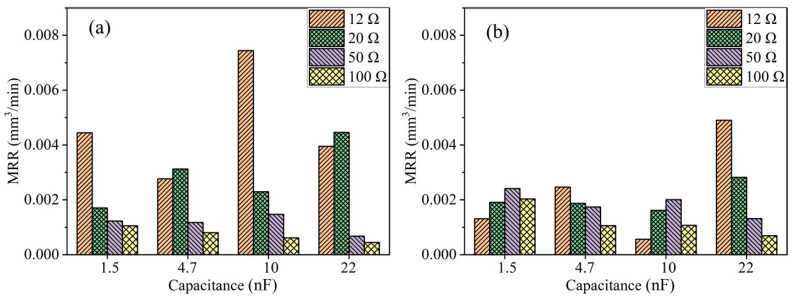
MRR of PCD at an open-circuit voltage of 200 V: (**a**) without ultrasonic vibration; (**b**) with ultrasonic vibration.

However, at an open-circuit voltage of 200 V, there is a significant improvement in the MRR, especially for smaller discharge resistances. As shown in Figure 6a, the MRR at discharge resistances of 12 Ω and 20 Ω is significantly higher than that at 50 Ω and 100 Ω. At such high open-circuit voltages, the resistance directly affects the machining efficiency. The high voltage implies greater discharge energies and larger inter-electrode gaps, making it easier for debris to be expelled. Additionally, due to the poor conductivity of PCD, it is less prone to arcing phenomena. Therefore, the enhancement in the open-circuit voltage is crucial for achieving such a significant improvement in MRR. At lower resistances, a relatively stable state can be achieved even without ultrasonic vibration. However, the addition of ultrasonic vibration may instead have an inhibitory effect. Figure 6b shows a bar chart of the MRR obtained with ultrasonic vibration, where, for most parameters, ultrasonic vibration does not lead to an improvement in the MRR. It is worth noting that at an open-circuit voltage of 200 V, except for the case with a discharge resistance of 12 Ω and a capacitance of 22 nF, ultrasonic vibration does not increase the MRR. Additionally, without ultrasonic vibration, the highest MRR occurs at a discharge resistance of 12 Ω and a capacitance of 10 nF. When introducing ultrasonic vibration, the MRR significantly decreases. This is because the original inter-electrode state has already reached stability, and the up-and-down vibration causes the electrode to vibrate near the optimal discharge position, thereby reducing the MRR. On the other hand, at higher resistances, the limited energy per discharge and slower downward die-sinking speed result in debris accumulating more easily. Therefore, introducing ultrasonic vibration at this point can effectively improve the gap environment and enhance MRR. In terms of electrode wear, taking the example of the combination of a 12 Ω resistor and four sets of capacitors (1.5 nF, 4.7 nF, 10 nF and 22 nF), the electrode wear in ultrasonic vibration-assisted die-sinking micro-EDM was measured at 45 μm, 29 μm, 12 μm and 15 μm respectively. Meanwhile, without ultrasonic vibration-assisted die-sinking micro-EDM, the electrode wear was recorded at 12 μm, 14 μm, 22 μm and 18 μm for the respective capacitor combinations. At 200 V, although the effect of ultrasound on MRR is minimal, there is observable electrode wear, suggesting that the relatively small change in MRR may be attributed to significant electrode wear.

**Figure 7:**
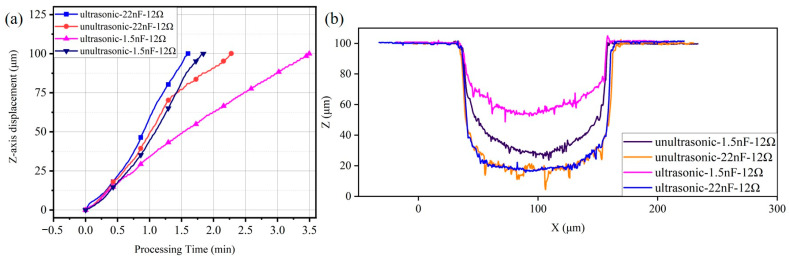
Machining trajectory and machined contours: (**a**) *z*-axis displacement trajectories during machining; (**b**) contours of the machined groove under an open-circuit voltage of 200 V.

In Figure 7a, the *z*-axis displacement trajectories under the machining parameters of an open-circuit voltage of 200 V, a capacitance of 22 nF and a discharge resistances of 12 Ω are presented. In the initial stages, the trajectories with and without ultrasonic vibration are almost identical. As the inter-electrode state gradually stabilizes, the machining speed increases. After reaching 25 μm, differences begin to emerge, as is the case with ultrasonic vibration maintaining its speed. On the contrary, in the case without ultrasonic vibration, the feeding rate starts to slow down. By the time the travel distance reaches 75 μm, the feeding rate of the case with ultrasonic vibration also begins to decelerate, but the machining efficiency remains significantly higher than that without ultrasonic vibration. The machining profiles with and without ultrasonic vibration -assistance at this energy level are shown in Figure 7b, where the machining depth and groove width are essentially the same, indicating that ultrasonic vibration has little impact on tool wear under these energy parameters and improves machining efficiency. As the machining depth grows, the cavitation effect becomes more pronounced.

However, under the machining parameters of an open-circuit voltage of 200 V, a capacitance of 1.5 nF and a discharge resistances of 12 Ω, ultrasonic vibration has an inhibitory effect. When the machining depth reaches 25 μm, the feeding rate with ultrasonic vibration significantly slows down and often experiences stopping before advancing. Because a capacitance of 1.5 nF results in a small amount of material removal with each discharge, die-sinking EDM without ultrasonic vibration can maintain a relatively stable state, allowing for slow progress. However, ultrasonic vibration disrupts this balanced state. In other words, the amplitude of ultrasonic vibrations may not be suitable for this low-energy condition. As shown in the curve for a capacitance of 1.5 nF in Figure 7, the machining profiles with and without ultrasonic vibration exhibit significant differences. The profile with ultrasonic vibration is noticeably shallower, indicating that the tool electrode contacts with the workpiece and secondary discharges occur frequently. This is one possible reason for the cessation of the trajectory.

### 3.2. Ultrasonic Vibration-Assisted Die-Sinking Micro-EDM of TA2

**Figure 8:**
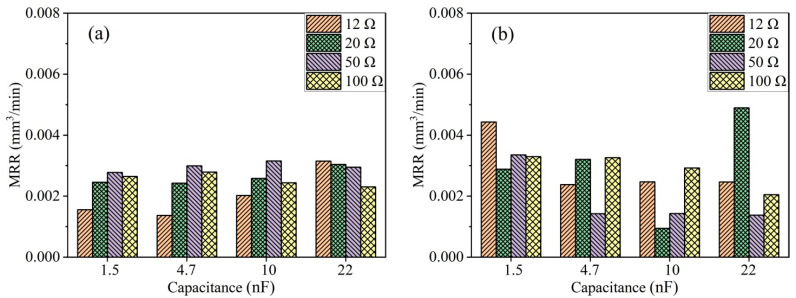
MRR of TA2 at different open-circuit voltages with ultrasonic vibration: (**a**) 100 V; (**b**) 200 V.

Due to the poor thermal conductivity of TA2, the energy generated by a single electrical discharge is difficult to diffuse, leading to the formation of arcing discharges. Die-sinking micro-EDM differs from EDM drilling in that the discharge electrode does not rotate at high speed, making it difficult to evacuate debris between the electrodes. This is one of the reasons why die-sinking micro-EDM struggles to machine titanium and its alloys [27]. The addition of ultrasonic vibration can effectively reduce arcing discharges. Figure 8 illustrates the bar chart of the MRR at open-circuit voltages of 100 V and 200 V for TA2. In Figure 8a, it is observed that at an open-circuit voltage of 100 V, when the discharge capacitance is small, resistances of 12 Ω and 20 Ω do not result in a higher MRR. Conversely, with an increase in resistance from 50 Ω to 100 Ω, the MRR of TA2 gradually increases. This is because at an open-circuit voltage of 100 V, the inter-electrode gap is small, and a small capacitance leads to a smaller amount of material removal per single discharge, while excessive discharge energy easily forms arcing discharges. However, with larger capacitance, the discharge energy becomes the primary factor influencing machining efficiency, not arcing discharges.

As shown in Figure 8b, the situation is more complex during ultrasonic vibration-assisted micro-EDM of TA2 at an open-circuit voltage of 200 V. In this case, most of the energy stored in the capacitance for the four resistance groups used in the experiment can be released. In this scenario, the main factors include the blocking effect of ultrasonic vibration on arcing discharges and the ability to clean the inter-electrode gap. The capacitance amount during micro-EDM of TA2 at 200 V has a significant impact on MRR. Compared to other capacitance groups, a smaller capacitance makes it less prone to arcing discharges, resulting in a higher MRR. However, for larger capacitance, the deionization time after each discharge is longer, leading to an overall lower MRR compared to the cases with smaller capacitance.

**Figure 9:**
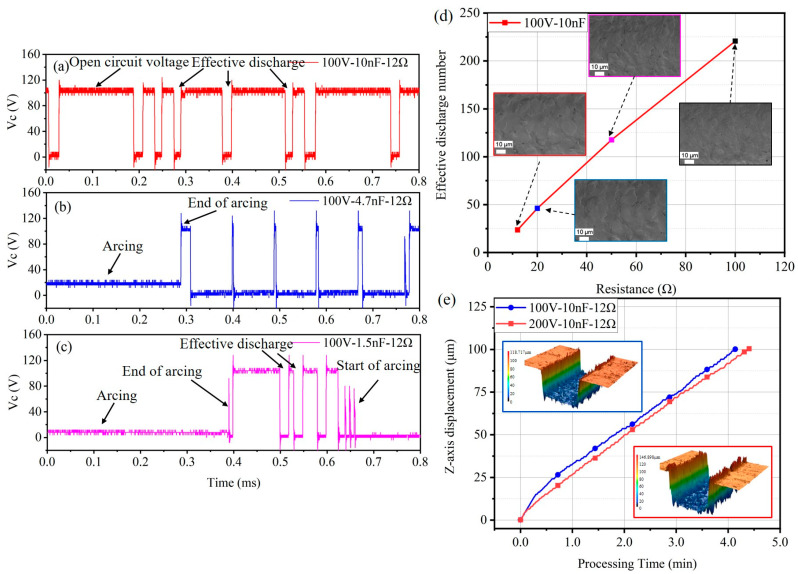
Discharge waveforms at different capacitance amounts: (**a**) 10 nF; (**b**) 4.7 nF; (**c**) 1.5 nF; (**d**) average effective discharge counts and SEM images at an open-circuit voltage of 100 V and a capacitance of 10 nF; (**e**) trajectories during ultrasonic vibration-assisted die-sinking micro-EDM of TA2.

As indicated by the MRR bar chart in Figure 8, the MRR during processing with a capacitance of 10 nF is higher than that with 1.5 nF and 4.7 nF capacitances. This result is also reflected in the discharge waveforms. Figure 9a–c shows the voltage waveforms during ultrasonic vibration-assisted die-sinking micro-EDM with different capacitances at an open-circuit voltage of 100 V and resistance of 12 Ω. During processing with a capacitance of 10 nF, the interval between individual discharges is long, resulting in a slow and relatively stable machining process with low discharge efficiency. For a capacitance of 4.7 nF, arcing phenomena frequently occur, and the cavitation effect breaks the arc. The monitored voltage can rise to 100 V, but it cannot maintain stable discharge after the next pulse discharge, leading to a peak-shaped voltage waveform. The overall discharge state tends to be arcing, resulting in low discharge efficiency. On the other hand, the discharge behavior with a capacitance of 1.5 nF exhibits a different pattern. After the cavitation effect breaks the arc, the monitored voltage shows a brief stable state. After several stable discharges, arcing phenomena reappear. Therefore, the overall discharge efficiency with a capacitance of 1.5 nF is also low and is comparable to the efficiency with a capacitance of 4.7 nF. Compared to machining PCD material, the wear when machining 100 μm TA2 is generally less, with electrode wear typically ranging from 3 to 10 μm. This is because the hardness of PCD material is much higher than that of TA2, making the tool less prone to wear.

As shown in Figure 9d, the average effective discharge counts and SEM images of ultrasonic vibration-assisted die-sinking micro-EDM of TA2 under different discharge resistances with an open-circuit voltage of 100 V and a capacitance of 10 nF are presented. The effective discharge counts in processing TA2 are positively correlated with discharge current, indicating that the cavitation effect has limited impact on breaking the arc. The smaller the discharge current, the easier it is to break the arc, returning the machining process to a normal discharge state. As seen in the SEM images, at a discharge resistance of 12 Ω, the machined bottom surface of TA2 exhibits sheet-like craters caused by arc discharge. Arc discharges lead to melting the surrounding workpiece at high temperatures, resulting in large areas of recast layer. With an increase in the discharge resistance, the sheet-like area on the machined surface becomes smaller. At a discharge resistance of 100 Ω, the average effective discharge count is 220, and the diameters of individual discharge craters are mostly below 10 μm. These results indicate that the cavitation effect on breaking the arc is influenced by the discharge resistance during processing of TA2. As the resistance increases and the current decreases, the effect of ultrasonic vibration on breaking the arc enhances, thereby affecting the effective discharge counts and surface quality.

Figure 9e shows the *z*-axis trajectories under open-circuit voltages of 100 V and 200 V, with a discharge capacitance of 10 nF and a discharge resistance of 12 Ω. In the initial stage, the trajectories of the two groups are close to overlapping, but in the subsequent 10 μm, the processing speed at an open-circuit voltage of 100 V is slightly faster than that at 200 V. From 25 μm to 50 μm, the feeding rates of both are almost the same, with some fluctuations observed at 75 μm. Finally, both groups process at almost the same speed up to 100 μm. In Figure 9e, the bottom surface craters at an open-circuit voltage of 200 V seem more likely to be caused by arcing discharge, presenting a sheet-like distribution rather than a crater shape. The groove at an open-circuit voltage of 200 V is wider, consistent with the pattern that a higher voltage results in a larger discharge gap. It is worth noting that the trajectories under both processing voltages do not exhibit long periods of tool retraction, further emphasizing the significant role of ultrasonic vibration in suppressing arcing discharge in ultrasonic vibration-assisted die-sinking micro-EDM.

### 3.3. Differential Behaviors of Ultrasonic Assistance on the Two Materials

**Figure 10:**
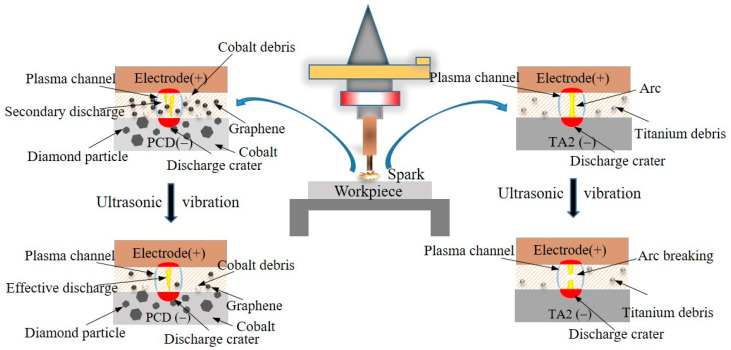
Schematic diagram of the inter-electrode gap state.

The PCD material, owing to its poor conductivity, is resistant to arcing phenomena even without the application of ultrasonic vibration. Conversely, TA2, due to its low thermal conductivity, faces challenges in the rapid dissipation of energy generated during die-sinking micro-EDM, leading to the easy formation of arcing discharges. Figure 10 shows the schematic diagram of discharge gaps of PCD and TA2 during ultrasonic vibration-assisted die-sinking micro-EDM. The discharge gap of PCD processing contains a lot of graphite, which facilitates secondary discharge. Subject to ultrasonic vibration, graphite and other discharge debris can be discharged in time to improve the discharge environment. For the case of TA2, due to the large processing area of the die-sinking micro-EDM process and the excellent adiabatic property of TA2, it is easy to pull the arc. The role of ultrasonic vibration at this time is to pull the arc off, so that the heat is brought out of the gap with the medium liquid. Through the above analysis, it can be observed that ultrasonic vibration-assisted die-sinking micro-EDM exhibits significant differences in processing these two materials. In this scenario, PCD faces significant disadvantages in terms of MRR and tool wear.

At low-energy conditions, ultrasonic vibration has a promoting effect on die-sinking micro-EDM of PCD. However, at higher voltages, where the discharge gap is larger, the discharge can maintain stable. In such cases, the addition of ultrasonic vibration may not significantly increase MRR and could even have inhibitory effects. On the contrary, for materials with poor thermal conductivity, such as TA2 in die-sinking micro-EDM, the introduction of ultrasonic vibration can be revolutionary in enhancing efficiency. Die-sinking micro-EDM of TA2 is primarily limited by arc discharge or, in other words, constrained by the conduction of discharge heat. In this scenario, the role of ultrasonic vibration mainly lies in breaking the electrical arc, improving the environment of the inter-electrode gap.

## 4. Conclusions

In this study, a series of experiments with various parameters were conducted on PCD and TA2 to investigate the impact of ultrasonic vibration on die-sinking micro-EDM of these two challenging materials. The key conclusions are as follows:

(1) At an open-circuit voltage of 100 V, ultrasonic vibration facilitates die-sinking micro-EDM of PCD. The promotion is particularly significant under a large capacitance and small discharge resistance, and the MMR can be increased by 3 to 5 times, with the maximum value reaching 0.0022 mm^3^/min. At an open-circuit voltage of 200 V, ultrasonic vibration promotes die-sinking micro-EDM for certain parameters but inhibits the process for the majority. In the case of TA2, whether at an open-circuit voltage of 100 V or 200 V, ultrasonic vibration demonstrates a promoting effect.
(2) The promoting effect of ultrasonic vibration on die-sinking micro-EDM differs fundamentally between PCD and TA2. For PCD materials, ultrasonic vibration facilitates continuous vertical movement of the electrode along the *z*-axis, accelerating the removal of discharge debris, while the cavitation effect of ultrasonic vibration improves the discharge environment between the electrodes. In the case of TA2, due to its poor thermal conductivity and susceptibility to arc discharge, the relative movement and cavitation effect generated by ultrasonic vibration can disrupt the arc, accelerating heat transfer.
(3) To maximize the promoting effect of ultrasonic vibration on die-sinking micro-EDM, it is necessary to adjust the amplitude of ultrasonic vibration in a timely manner to adapt different die-sinking depths, different energy levels and different materials. For large discharge energies, especially when the material has poor thermal conductivity, increasing the ultrasonic amplitude is advisable. For small discharge energy, or when machining materials with a high melting point and good thermal conductivity, reducing the amplitude is recommended. However, it is important to note that this study has some limitations, such as not fully investigating the effect of ultrasonic vibration on tool wear. These limitations provide directions for future research to further expand the understanding and application of this field.

## Data Availability

The original contributions presented in the study are included in the article, further inquiries can be directed to the corresponding author.
